# The purchase sources of and price paid for cigarettes in six European countries: Findings from the EUREST-PLUS ITC Europe Surveys

**DOI:** 10.18332/tid/100413

**Published:** 2019-03-26

**Authors:** Tibor Demjén, Judit Kiss, Piroska A. Kovács, Ute Mons, Sarah Kahnert, Pete Driezen, Christina N. Kyriakos, Mateusz Zatoński, Krzysztof Przewoźniak, Marcela Fu, Esteve Fernández, Ann McNeill, Marc Willemsen, Yannis Tountas, Antigona C. Trofor, Geoffrey T. Fong, Constantine I. Vardavas, on behalf of the EUREST-PLUS consortium

**Affiliations:** 1 Smoking or Health Hungarian Foundation (SHHF), Budapest, Hungary; 2 Cancer Prevention Unit and WHO Collaborating Centre for Tobacco Control, German Cancer Research Center (DKFZ), Heidelberg, Germany; 3 Medical Faculty, Heidelberg University, Heidelberg, Germany; 4 Department of Psychology, University of Waterloo (UW), Waterloo, Canada; 5 European Network for Smoking and Tobacco Prevention (ENSP), Brussels, Belgium; 6 University of Crete (UoC), Heraklion, Greece; 7 Health Promotion Foundation (HPF), Warsaw, Poland; 8 European Observatory of Health Inequalities, President Stanisław Wojciechowski State University of Applied Sciences, Kalisz, Poland; 9 Maria Skłodowska-Curie Institute-Oncology Center (MSCI), Warsaw, Poland; 10 Catalan Institute of Oncology (ICO), University of Barcelona, Barcelona, Spain; 11 Bellvitge Biomedical Research Institute (IDIBELL), Barcelona, Spain; 12 UK Centre for Tobacco and Alcohol Studies, King’s College London (KCL), London, United Kingdom; 13 Department of Health Promotion, Maastricht University, Maastricht, the Netherlands; 14 Netherlands Expertise Center for Tobacco Control (Trimbos Institute), Utrecht, the Netherlands; 15 National and Kapodistrian University of Athens (UoA), Athens, Greece; 16 University of Medicine and Pharmacy ‘Grigore T. Popa’ Iasi, Iasi, Romania; 17 Aer Pur Romania, Bucharest, Romania; 18 Ontario Institute for Cancer Research, Toronto, Canada; 19 School of Public Health and Health Systems, University of Waterloo (UW), Waterloo, Canada

**Keywords:** purchase sources of tobacco products, reducing the number of purchase sources, price paid for tobacco

## Abstract

**INTRODUCTION:**

Tobacco tax policies have been proven to be effective in reducing tobacco consumption, but their impact can be mitigated through price-minimizing behaviours among smokers. This study explored the purchase sources of tobacco products and the price paid for tobacco products in six EU member states.

**METHODS:**

Data from Wave 1 of the EUREST-PLUS ITC Europe Survey collected from nationally representative samples of adult smokers in Germany, Greece, Hungary, Poland, Romania and Spain (ITC 6E Survey) were used. The ITC 6E Survey sample, conducted in 2016, randomly sampled 6011 adult cigarette smokers aged 18 years or older. Information on purchase sources of tobacco was examined by country. The difference in reported purchase price by purchase location (store vs non-store/other) was analysed using linear regression for each country.

**RESULTS:**

Tobacco purchasing patterns and sources varied widely between countries. Non-store/other purchases were very rare in Hungary (0.1%) while these types of purchases were more common in Germany (5.1%) and Poland (8.6%). Reported prices of one standard pack of 20 cigarettes were highest in Germany (4.80€) and lowest in Hungary (2.45€). While non-store purchases were only made by a minority of smokers (>10% in all countries), the price differential was considerable between store and non-store/other sources, up to 2€ per pack in Greece and in Germany.

**CONCLUSIONS:**

The results suggest a huge variation of purchasing sources and price differentials between store and non-store purchasing sources across the six EU member states examined. While the cross-sectional data precludes any causal inference, supply chain control through licensing as introduced in Hungary and the lack of such measures in the other countries might nevertheless be a plausible explanation for the large differences in the frequency of non-store purchases observed in this study.

## INTRODUCTION

Smoking is the largest avoidable risk for premature death in the European Union (EU), responsible for 0.7 million deaths each year^1^. Approximately half of smokers die prematurely, resulting in an average loss of 10 years of life^2^. However, high smoking rates persist with one in four EU citizens still smoking^3^. Raising cigarette prices through tobacco taxes is considered to be the single most effective intervention to reduce demand for cigarettes^4,5^. According to a recent simulation study^6^, an average price increase of 10% throughout the EU would lead to an average increase in revenues by 6.8% and the average tobacco taxation benefit of all EU countries would increase by US$ 6.6 million. Most importantly, such a rise in cigarette price would significantly reduce cigarette consumption and the number of deaths caused by smoking^6^.

The World Health Organization (WHO) Framework Convention on Tobacco Control (FCTC) Article 6 includes the recommendation that each Party implements taxation and pricing policies to reduce the demand for tobacco, taking into account the sovereign right of the Parties to determine and establish their own taxation policies^7^. Fifty of 53 countries in the WHO European region have ratified the WHO FCTC^8^ including all countries that participate in the EUREST-PLUS project. In the EU, regulations of the Council Directive 2011/64/EU provide for a minimum amount of excise duty of 90€ per 1000 cigarettes and minimum excise duties of 60% of the weighted average retail price (unless excise tax is higher than €115/1000 cigarettes)^9^. WHO recommends a tax rate of at least 75% of cigarette retail prices, which — based on EU reported rates from July 2016 — is achieved by 26 of the 28 EU member states^10^. However, considerable price differentials between different tobacco products as well as between countries remain, providing smokers with cheaper alternatives and potentially weakening the health impact of tax policies.

Previous studies on purchasing patterns have shown that significant numbers of smokers engage in cost-reducing tobacco purchasing behaviours in several countries^11-14^. Given the scarcity of comparative studies of tobacco purchasing patterns in the EU thus far, the purpose of this study was to explore purchasing behaviours, i.e. where smokers buy tobacco and the price of tobacco products, in six EU countries.

## METHODS

### Study design

This study was conducted in the framework of the EUREST-PLUS project (European Regulatory Science on Tobacco: Policy implementation to reduce lung diseases)^15^. One objective of EURESTPLUS is to monitor and evaluate the impact of tobacco control policies through the creation of a longitudinal cohort of adult smokers in 6 EU countries (Germany, Greece, Hungary, Poland, Romania and Spain). This cohort study—the EUREST-PLUS ITC Europe (ITC 6E) Survey—is part of the ongoing International Tobacco Control (ITC) Policy Evaluation Project^16^.

Data were collected between June and September 2016 from nationally representative samples of adult cigarette smokers aged 18 years or older in six EU countries (Germany: n=1003; Greece: n=1000; Hungary: n=1000; Poland: n=1006; Romania: n=1001; Spain: n=1001; Total n=6011). The geographic strata were NUTS (Nomenclature of Territorial Units for Statistics) regions crossed with degree of urbanization (urban, intermediate, rural). Approximately 100 area clusters were sampled in each country, with the aim of obtaining 10 adult smokers per cluster. Clusters were allocated to strata proportionally to the size of the population aged 18 years and older. Within each cluster, household addresses were sampled using a random walk design. Where possible, one randomly selected male smoker and one randomly selected female smoker were chosen for interview from a sampled household. Screening of households continued until the required number of smokers from the cluster had been interviewed. All interviews were conducted face-to-face by interviewers using tablets (CAPI). Further details on methods used in the ITC 6E Survey can be found elsewhere^17^.

### Ethics procedures

The study was approved by the Research Ethics Board of the University of Waterloo, Ontario, Canada and by local ethics boards within the study countries. Participation in the study was contingent on provision of individual informed consent, which was obtained either in written or verbal form according to local ethical requirements. The EUREST-PLUS Project is registered in Clinicaltrials.gov with trial registration number NCT02773836.

### Measures

Respondents were asked about the last time they purchased their tobacco products and the purchase source, the product purchased, and the price they paid. The purchase sources were ascertained by asking ‘Where did you last buy cigarettes or tobacco for yourself?’ with response options: ‘Large grocery store/supermarket’, ‘Small grocery store/convenience store’, ‘Bar, restaurant, or entertainment establishment’, ‘Duty-free shop (airport or boat terminal)’, ‘From someone else selling cigarettes independently and/or illegally’, ‘The internet’, ‘Newsstand’, ‘Tobacconist’, ‘Vending machine’, ‘Kiosk’, ‘Gas station’, and ‘Other’. For some analyses these purchase sources were categorized into ‘store’ vs ‘non-store/other’, for which the options: ‘Duty-free shop’, ‘From someone else selling cigarettes independently and/or illegally’, and ‘The internet’ and ‘Other/don’t know’ where categorized as ‘non-store/other’, and all other options were collapsed into the ‘store’ category. For the product purchased, respondents were asked whether the last time they bought cigarettes for themselves they purchased: ‘A carton of factory-made cigarettes’, ‘A pack of factory-made cigarettes’, ‘Rolling tobacco’ or ‘Both packages of factory-made cigarettes and rolling tobacco’. The respondents were then asked to indicate the price they paid per carton or pack of factory-made (FM) cigarettes, as well as the size of the carton (number of packs) and/or pack (in number of cigarettes), or the price they paid for roll-your-own (RYO) tobacco, respectively, and the size of the RYO unit (in grams). Based on this information, the price per cigarette was calculated. To obtain prices for RYO, it was assumed that 0.75 g of RYO was equivalent to 1 cigarette^18^. Prices per cigarette or cigarette equivalent were then multiplied by 20 to obtain the price per standard pack of 20 cigarettes. All prices were converted to Euros for Hungary, Poland and Romania, based on historical exchange rates from July (using the online currency converter http://www.xe.com).

Respondents were described according to sociodemographic characteristics (sex, age group, degree of urbanization, highest level of education, monthly household income), and smoking behaviour. Age was categorized into four age groups (18–24, 25–39, 40–54, and 55 years and older). Degree of urbanization was classified as rural, intermediate or urban. Education was categorized into low (pre-primary, primary, lower secondary), moderate (upper secondary, post-secondary non-tertiary, short-cycle tertiary), and high (bachelor or equivalent, master or equivalent, doctoral or equivalent). Monthly household income information was collected using local currencies and was used to classify respondents into low, moderate or high income using country specific thresholds (Germany: <1750€, 1750 to <3000€, ≥3000€; Greece: <750€, 750 to <1500€, ≥1500€; Hungary: ≤150000 Ft, 150001 to ≤250000 Ft, >250000 Ft; Poland: ≤2000 zl, 2001 to 4000 zl, > 4000 zl; Romania: ≤1000 lei, 1001 to 2500 lei, >2500 lei; Spain: <1250€, 1250 to <2500, ≥2500€). Smoking behaviour was described by frequency of smoking (daily, weekly or monthly), smoking intensity measured as number of cigarettes smoked per day (≤10, 11–20, 21–30, ≥31), and type of cigarettes smoked (FM only, RYO only, or both).

### Statistical analysis

Purchase patterns and price paid were examined separately by country with cross tabulations, using the Rao-Scott chi-squared test or t-test, respectively, to test for statistically significant differences across countries. In an analysis using pooled data, a country by purchase location interaction term was included to test for differences across countries. The difference in reported purchase price by country and purchase location was analysed using a linear regression model for each country. Data were analysed using SAS 9.4. Data were weighted to ensure results were representative of the population of smokers in each of the 6 EU countries. All statistical analysis accounted for the complex sampling design of the ITC 6E Survey.

## RESULTS

The sociodemographic characteristics of the sample of smokers in each country are shown in Table 1. The sex distribution of respondents was similar across countries, with the exception of Romania, where 58% of sampled smokers were male. In each country, fewer than 12% of respondents were aged 18–24 years, while similar percentages were aged 25–39, 40–54 or 55 years and older. The degree of urbanization differed across countries: 53% of respondents from Spain lived in urban areas compared to only 17% of respondents in Greece. The proportion of rural respondents was the highest in Romania (40%) and lowest in Spain (15%). The educational level of respondents showed relevant differences in the countries surveyed: the highest proportion of low educational level was in Hungary (62%), the highest proportion of moderate educational level was seen in Poland (76%), and the highest proportion of high educational level in Greece (20%). Based on the monthly household income, the respondents had a diverse background: the proportion of respondents with low monthly incomes was highest in Germany (30.5%); the proportion of respondents with middle-level monthly income was highest in Greece (52.5%), while the proportion of high-income respondents was lowest in Spain (7%). It is noteworthy that more than 30% of respondents from Hungary, Poland and Spain refused to answer this question.

**Table 1:** Characteristics of smokers participating in Wave 1 of the EUREST-PLUS ITC Survey (n=6011)^a^

	*Germany n (%)*	*Greece n (%)*	*Hungary n (%)*	*Poland n (%)*	*Romania n (%)*	*Spain n (%)*
**Sex**						
Male	507 (50.5)	544 (54.4)	521 (52.1)	477 (47.4)	581 (58.0)	545 (54.4)
Female	496 (49.5)	456 (45.6)	479 (47.9)	529 (52.6)	420 (42.0)	456 (45.6)
**Age group**						
18–24	88 (8.8)	61 (6.1)	59 (5.9)	72 (7.2)	110 (11.0)	117 (11.7)
25–39	283 (28.2)	255 (25.5)	282 (28.2)	342 (34.0)	300 (30.0)	312 (31.2)
40–54	339 (33.8)	383 (38.3)	357 (35.7)	281 (27.9)	321 (32.1)	323 (32.3)
≥55 years	293 (29.2)	301 (30.1)	302 (30.2)	311 (30.9)	270 (27.0)	249 (24.9)
**Degree of urbanization**						
Urban	380 (37.9)	170 (17.0)	342 (34.2)	341 (33.9)	360 (36.0)	532 (53.1)
Intermediate	409 (40.8)	609 (60.9)	368 (36.8)	309 (30.7)	240 (24.0)	320 (32.0)
Rural	214 (21.3)	221 (22.1)	290 (29.0)	356 (35.4)	401 (40.1)	149 (14.9)
**Education**						
Low	509 (50.8)	306 (30.7)	617 (61.8)	123 (12.4)	246 (24.9)	410 (41.0)
Moderate	417 (41.7)	488 (48.9)	311 (31.2)	753 (76.1)	629 (63.6)	506 (50.7)
High	75 (7.5)	203 (20.4)	70 (7.0)	114 (11.5)	114 (11.5)	83 (8.3)
**Household income**						
Low	306 (30.5)	180 (18.0)	179 (17.9)	173 (17.2)	226 (22.6)	271 (27.1)
Moderate	347 (34.6)	525 (52.5)	290 (29.0)	353 (35.1)	466 (46.6)	268 (26.8)
High	257 (25.6)	98 (9.8)	220 (22.0)	154 (15.3)	250 (25.0)	68 (6.8)
Not reported	93 (9.3)	197 (19.7)	311 (31.1)	326 (32.4)	59 (5.9)	394 (39.4)

a The data shown are unweighted.

Table 2 gives the smoking characteristics of smokers across the 6 EU countries. Differences were tested across countries using the Rao-Scott chi-squared test; the differences presented were statistically significant (p<0.001). With the exception of Germany (88%), more than 95% of smokers from each country smoked on a daily basis. Most smokers reported smoking more than 10 cigarettes a day; Greece had the highest proportion of heavy smokers (25% smoked more than 20 cigarettes/day), while Poland had the lowest (10%). On average, Greek smokers reported smoking 20 cigarettes/day compared to only 15 cigarettes/day among German smokers.

**Table 2:** Smoking characteristics of the 6 EU countries (N=6011)

	*Germany*		*Greece*		*Hungary*		*Poland*		*Romania*		*Spain*	
	*%*	*(95% CI)*	*%*	*(95% CI)*	*%*	*(95% CI)*	*%*	*(95% CI)*	*%*	*(95% CI)*	*%*	*(95% CI)*
**Frequency of smoking**												
Daily	88.3	(84.4–91.4)	96.9	(94.3–98.5)	98.9	(97.8–99.6)	96.4	(94.9–97.5)	94.8	(92.5–96.5)	97.2	(95.5–98.3)
Weekly	9.3	(6.8–12.5)	2.6	(1.1–5.0)	0.7	(0.3–1.5)	3.1	(2.0–4.5)	4.5	(3.0–6.5)	1.4	(0.7–2.3)
Monthly	2.4	(1.2–4.4)	0.5	(0.1–1.4)	0.4	(0.0–1.4)	0.6	(0.2–1.4)	0.7	(0.1–2.2)	1.5	(0.7–2.8)
**Cigarettes/day**												
≤10	36.6	(31.8–41.8)	28.5	(24.9–32.4)	30.0	(26.1–34.2)	31.2	(27.5–35.2)	32.5	(28.9–36.3)	40.1	(35.9–44.4)
11–20	49.2	(44.4–54.1)	46.4	(42.5–50.5)	58.7	(55.1–62.3)	58.6	(54.1–62.9)	55.9	(52.2–59.5)	48.1	(43.9–52.3)
21–30	10.5	(8.3–13.1)	12.4	(10.1–15.2)	8.5	(6.4–11.3)	7.8	(5.6–10.9)	6.9	(5.1–9.3)	7.2	(5.5–9.4)
≥31	3.7	(2.4–5.5)	12.6	(9.9–16.1)	2.8	(1.7–4.3)	2.4	(1.4–3.7)	4.7	(3.0–7.0)	4.6	(3.1–6.5)
**Smoking factory-made cigarettes or roll-your-own cigarettes**												
Factory-made (FM) cigarettes only	73.0	(67.6–77.8)	70.5	(64.8–75.7)	45.8	(40.3–51.4)	80.1	(75.2–84.3)	93.7	(91.3–95.4)	72.6	(68.4–76.5)
Roll-your-own (RYO) cigarettes only	11.4	(8.5–15.1)	27.3	(22.8–32.3)	45.8	(40.7–51.0)	7.9	(5.5–11.3)	0.9	(0.3–2.0)	17.5	(14.0–21.8)
Both	15.6	(12.2–19.7)	2.2	(1.1–3.9)	8.4	(6.1–11.5)	12.0	(8.9–15.9)	5.4	(3.8–7.6)	9.8	(7.8–12.3)
**Last purchased**												
Carton	11.1	(8.5–14.5)	1.2	(0.6–2.1)	4.0	(2.4–6.1)	5.2	(3.4–7.7)	6.0	(4.4–8.1)	6.0	(4.6–8.0)
Pack	69.4	(64.7–73.8)	71.0	(65.5–76.0)	47.5	(41.4–53.7)	83.6	(79.6–87.0)	91.5	(88.5–93.8)	71.7	(67.1–75.9)
Rolling tobacco	15.4	(12.0–19.7)	27.8	(22.8–33.3)	46.3	(40.3–52.4)	10.8	(7.9–14.6)	1.5	(0.6–3.3)	20.4	(16.9–24.3)
Both FM + RYO packages	4.0	(2.4–6.3)	0.1	(0.0–0.5)	2.2	(1.1–3.9)	0.4	(0.1–1.2)	1.0	(0.3–2.3)	1.9	(0.9–3.4)

Except for Hungary (46%), more than 70% of smokers in each country smoked FM with the high proportions in Romania (94%) and Poland (80%). Hungary had the highest proportion smoking RYO (46%), followed by Greece (27%), Spain (18%) and Germany (11%) (Rao-Scott chi-squared test p<0.001).

With the exception of Hungary, most smokers reported last purchasing FM cigarettes by the pack (from 69% of German smokers to 92% of Romanian smokers). In Hungary, 48% of smokers last purchased FM cigarettes by the pack while 46% last purchased RYO. Carton purchases were relatively rare in most countries (varying from 1% in Greece to 4–6% in Hungary, Poland, Romania and Spain), except for Germany, where 11% of smokers reported last purchasing cigarettes by the carton.

Table 3 presents the last purchase sources of tobacco among the smokers of the 6 EU countries. Differences by purchase location (store vs non-store/ other) varied by country (Rao-Scott chi-squared test p<0.001). Non-store/other purchases were rare in Hungary (only 0.1% of smokers made such purchases) while these types of purchases were somewhat more common in Germany (5.1% of smokers reported such purchases) and Poland (8.6%). The illegal forms of purchasing tobacco products were at the lowest level in Hungary (0%) and highest in Poland (5.4%). Smokers in Hungary (98%) and Spain (76%) mostly acquired tobacco products from tobacco shops (tobacconists), from kiosks in Greece (71%), and from convenience stores in Poland (60%) and Romania (53%). In Germany, most smokers bought tobacco products from supermarkets (34%) and gas stations (24%).

**Table 3:** The last purchase sources of tobacco products (‘Where did you last buy cigarettes or tobacco for yourself?’) across the 6 EU countries (N=6011)

	*Germany (n=1003)*		*Greece (n=1000)*		*Hungary (n=1000)*		*Poland (n=1006)*		*Romania (n=1001)*		*Spain (n=1001)*	
*Last Purchase Source*	*%*	*(95% CI)*	*%*	*(95% CI)*	*%*	*(95% CI)*	*%*	*(95% CI)*	*%*	*(95% CI)*	*%*	*(95% CI)*
**Store^a^**	94.9	(91.3–97.1)	98.4	(97.0–99.3)	99.9	(99.4–100.0)	91.4	(88.0–93.9)	96.4	(94.5–97.8)	95.5	(92.8–97.4)
Large grocery store/ supermarket	34.2	(29.5–39.2)	2.9	(0.8–7.2)	1.2	(0.1–4.5)	13.3	(9.9–17.6)	35.2	(29.2–41.6)	0.5	(0.1–2.0)
Small grocery store/ convenience store	2.8	(1.5–4.6)	21.8	(15.0–30.6)	0.8	(0.2–2.4)	60.1	(54.6–65.3)	52.6	(45.9–59.2)	0.5	(0.1–1.4)
Bar/restaurant/ entertainment establishment	0.2	(0.0–0.7)	1.0	(0.0–5.7)	0.1	(0.0–0.5)	0.3	(0.0–1.1)	1.8	(0.9–3.3)	9.4	(6.9–12.7)
Newsstand	3.7	(2.5–5.2)	0.2	(0.0–0.9)	0	–	1.8	(0.7–3.7)	0.1	(0.0–0.7)	0.6	(0.2–1.3)
Tobacconist	13.7	(10.7–17.5)	0.9	(0.3–1.9)	97.8	(93.7–99.5)	1.8	(0.8–3.3)	0.5	(0.1–1.2)	75.5	(71.6–79.0)
Vending machine	6.1	(4.4.–8.5)	0	–	0	–	0	–	0	–	7.5	(5.0–11.3)
Kiosk	10.5	(7.3–14.7)	71.3	(62.4–78.9)	0	–	11.8	(8.8–15.7)	3.1	(1.7–5.2)	1.1	(0.3–3.0)
Gas station	23.7	(20.3–27.5)	0.3	(0.0–1.0)	0	–	2.3	(1.2–3.9)	3.1	(1.7–5.1)	0.3	(0.1–1.0)
**Non-store/other^a^**	5.1	(2.9–8.7)	1.6	(0.7–3.0)	0.1	(0.0–0.6)	8.6	(6.1–12.0)	3.6	(2.2–5.5)	4.5	(2.6–7.2)
Duty-free	0.6	(0.2–1.3)	0.3	(0.1–0.9)	0	–	0	–	0.2	(0.0–0.8)	0.2	(0.0–1.3)
Someone else selling independently/illegally	1.6	(0.2–5.4)	1.2	(0.4–2.5)	0	–	5.4	(3.2–8.7)	1.5	(0.7–2.9)	2.2	(0.9–4.5)
The internet	0.2	(0.0–0.7)	0	–	0	–	0.4	(0.0–1.5)	0.1	(0.0–0.8)	0.6	(0.2–1.4)
Other/don't know	2.7	(1.3–5.0)	0.1	(0.0–0.6)	0.1	(0.0–0.6)	2.9	(1.7–4.6)	1.7	(0.8–3.2)	1.4	(0.5–3.0)

a Store vs non–store/other purchases differ by country (Rao–Scott chi–squared test = 52.84, df = 5, p<0.001)

Table 4 shows estimated price by country and purchase location as well as the difference between store/non-store purchases within each country. Although few non-store purchases were reported in Hungary, due to missing data for purchase price, there were no respondents left in that particular cell, so prices by non-store/other purchase locations in Hungary could not be estimated. There were significant differences between the average prices of tobacco products by last purchase location (store vs non-store) in four of the six countries (Germany, Greece, Romania and Spain) (t-test p<0.001). The average price difference between the tobacco products bought in stores compared to non-store locations was highest in Greece (1.96€, p<0.001) and Germany (1.95€, p<0.001). There was no difference in average purchase price in Poland (0.5€, p=0.497). The average price of tobacco products purchased from stores was highest in Germany (4.80€) while the average price of tobacco products bought in stores was lowest in Hungary (2.45€). The lowest price of non-store tobacco products was observed in Greece (1.76€).

**Table 4:** The average price/pack by the last purchase sources (store/non-store) of tobacco products in the 6 EU countries

*Country*	*Last purchase source*									
	*Store*			*Non-store/other*			*Difference*		*Test*	
	*Average Price^a^*			*Average Price^a^*			*Average Price^a^*			
	*(n)*	*Mean*	*(95% CI)*	*(n)*	*Mean*	*(95% CI)*	*Mean*	*(95% CI)*	*t*	*p*
Germany	(923)	4.80	(4.60–5.0)	(46)	2.85	(2.49–3.21)	1.95	(1.57–2.32)	10.23	<0.001
Greece	(950)	3.72	(3.63–3.8)	(18)	1.76	(1.45–2.06)	1.96	(1.64–2.28)	11.92	<0.001
Hungary	(947)	2.45	(2.27–2.64)	–	–	–	–	–	–	–
Poland	(889)	2.98	(2.75–3.22)	(76)	2.47	(1.03–3.92)	0.51	(0.96–1.97)	0.68	0.497
Romania	(955)	3.24	(3.19–3.3)	(40)	2.10	(1.60–2.59)	1.15	(0.66–1.64)	4.61	<0.001
Spain	(918)	3.94	(3.82–4.06)	(35)	2.31	(1.62–3.01)	1.63	(0.95–2.32)	4.67	<0.001

a Reported prices are in € per standard pack of 20 cigarettes. Purchases of rolling tobacco were converted to grams needed to roll 1 cigarette (0.75 g) and then multiplied by 20 to obtain price per 20 cigarette equivalents.

## DISCUSSION

This is the first study that explores and compares cigarette purchasing patterns in several EU member states. This study of smokers from six EU member states showed large differences with regards to cigarette purchasing patterns between countries. While the vast majority of tobacco purchases were made in stores, the types of stores differed across countries with tobacconists being the most used source in Hungary and Spain compared with kiosks in Greece, convenience stores in Poland and Romania, and a wide range of sources in Germany with large stores being the most common. Illicit sources were generally rare, with no purchases from illegal sources in Hungary, and most common in Poland.

There were significant differences between the average prices of tobacco products by last purchase location (store vs non-store) in four of the six countries. The average price of tobacco products purchased from stores was highest in Germany and was lowest in Hungary. The average prices from store-sources were roughly in line with the weighted average prices reported by the EU^10^. While non-store purchases were only made by a small minority of smokers (<10% in all countries), the price differential was found to be considerable. The average absolute price difference between the tobacco products bought in stores compared to non-store locations was highest in Greece and Germany, with about 2€ difference per pack.

The findings of this study are consistent with studies using comparable ITC data from other countries showing significant portions of smokers purchasing cheaper forms of tobacco such as RYO, and/or purchasing from low-cost or tax-free sources, such as in China^12^, United Kingdom^14^, and the United States^11^. There is evidence that smokers mitigate price increases through purchasing behaviour: studies indicate that smokers make use of available options to reduce their tobacco expenditures^11,12,14^, while changes in purchase patterns seem to be related to price increases^11,14^. This has important public health implications as purchasers of low/untaxed cigarettes tend to make less quit attempts compared to purchasers of full-priced cigarettes^13^.

Even though reduction in the number of legal retail outlets is not mentioned in the WHO FCTC as a measure to influence demand for tobacco, controlling the supply chain is nevertheless seen as important for efficient and effective tax administration^19^. For example, Hungary implemented a law in 2012 prohibiting tobacco sales other than in national tobacco shops. Due to this law, from 1 July 2013, tobacco products can be purchased only in 7000 controlled stores, instead of more than 40000 shops in the country earlier. While the cross-sectional nature of this study precludes any causal interpretation, this law could nevertheless be a likely explanation for the extremely high prevalence of cigarette purchases at tobacconists and the very low non-store purchases observed in Hungary. Rather than using lower-cost or illicit sources, price-minimizing behaviours in Hungary might be reflected in the comparably high prevalence of purchases of cheaper RYO.

Tobacco taxation has been recognized as the most effective tool to curb smoking^5,20^. WHO recommends a simple tax system whereby excise taxes on all tobacco products represent a minimum of 75% of the retail price^21^. However, as higher prices give smokers the incentive to look for cheaper tobacco products (e.g. RYO) and brands, it is essential to reduce the difference between the prices of different products and brands. In addition, measures should be taken to combat illicit trade and to control the supply chain. This would not only ensure that taxation measures unfold their full public health impact but also would make tobacco tax revenues reliable and stable.

### Strengths and limitations

The strength of this study is that large samples of current smokers with internationally standardized questionnaires could be analysed to explore tobacco purchase patterns. The cross-sectional nature of the data however precludes any causal inference, especially with regards to the impact of policies on prices paid or purchase sources. Some caution is also warranted regarding the validity of the data because the self-reported measures are prone to recall and social desirability bias. In particular, the use of illicit sources of tobacco could be underreported. Future studies using longitudinal data should further examine changes in prices or purchase sources, in relation to smoking behaviour and cessation.

## CONCLUSIONS

The results of this study suggest large variations in purchasing sources between countries as well as price differentials between store and non-store tobacco purchases across the six EU member states examined. RYO use also varied across countries. Due to the lower tax levels of RYO tobacco, smokers in this study may have reduced their tobacco expenditures by choosing to smoke RYO tobacco instead of factory-made cigarettes. To ensure that national price and tax policies yield maximal potential, countries should harmonize tax rates across tobacco products. Smokers also purchase tobacco from illicit tax-free or low-cost sources. Even though non-store purchases were rare in all countries, price differentials between store and non-store purchases were considerable. Low-cost non-store purchases can undermine national tax and price policies. While the cross-sectional study design does not allow any causal inferences to be made, the results are nevertheless consistent with the interpretation that Hungary’s strategy to implement a system of national retailer licensing may have prevented non-store purchases at potentially lower prices.
